# Radiographic evidence of metaphyseal sclerosis secondary to canine distemper virus: 4 cases in juvenile dogs

**DOI:** 10.1111/jvim.16453

**Published:** 2022-06-03

**Authors:** Kryssa L. Johnson, Linden E. Craig, Sabrina Wilson, Ehren McLarty, Adrien‐Maxence Hespel

**Affiliations:** ^1^ Department of Radiology and Diagnostic Imaging College of Veterinary Medicine, University of Tennessee Knoxville Tennessee USA; ^2^ Department of Biomedical and Diagnostic Sciences College of Veterinary Medicine, University of Tennessee Knoxville Tennessee USA; ^3^ Diagnostic Imaging Service, School of Veterinary Medicine University of California, Davis Davis California USA

**Keywords:** bone, infectious disease, pathology, puppy, radiology, skeletal

## Abstract

**Background:**

Metaphyseal sclerosis secondary to canine distemper virus has been described histopathologically, but its radiographic appearance has not been described.

**Objectives:**

Describe the radiographic appearance of metaphyseal sclerosis secondary to canine distemper virus in juvenile dogs as distinct from metaphyseal osteopathy (formerly called hypertrophic osteodystrophy).

**Animals:**

Four dogs (2 intact females and 2 intact males) between 2.5 and 4 months of age presented to 2 different veterinary teaching hospitals.

**Methods:**

Retrospective case series in which definitive diagnosis of canine distemper virus based on antemortem positive reverse transcription‐polymerase chain reaction (RT‐PCR) result or necropsy was required.

**Results:**

All 4 dogs were presented for evaluation of neurologic abnormalities, respiratory signs, and lethargy; 2 dogs had gastrointestinal signs and ocular abnormalities. Radiographs on all patients featured multifocal, symmetric, metaphyseal sclerosis, with no evidence of lysis or changes to the adjacent growth plate. The metaphyseal sclerosis was most apparent at the proximal humeral diaphyses and other included long bones. Diagnosis of distemper was confirmed by necropsy (2 of 4 dogs) or positive RT‐PCR results (2 of 4 dogs). Three dogs were euthanized because of progressive illness, and 1 dog was lost to follow‐up.

**Conclusion and Clinical Importance:**

Identification of metaphyseal sclerosis on radiographs during diagnostic evaluation of young dogs should lead to a clinical suspicion of canine distemper virus infection. Sclerosis identified secondary to canine distemper virus is distinct from the necrosis and inflammation of metaphyseal osteopathy.

**Abbreviations**
CDV: canine distemper virus
HCT: hematocrit
RBC: red blood cells
RT‐PCR: reverse transcriptase polymerase chain reaction
WBC: white blood cells

## 1 INTRODUCTION

Canine morbillivirus (canine distemper virus [CDV]) produces a wide range of clinical findings, including fever, lethargy, inappetence, vomiting, diarrhea, respiratory signs, conjunctivitis, neurologic signs, and hyperkeratosis of the footpads and nasal planum.^1^, ^2^ A diagnosis of CDV is not always straightforward and usually is based on clinical signs, physical and neurologic examination findings, or both, and additional diagnostic test results. The most commonly utilized tests to definitively diagnose CDV are RT‐PCR or histopathology.^1^, ^2^ The RT‐PCR tests can be performed on a variety of body fluid or tissue samples, including blood, urine, respiratory secretions, conjunctival swabs, or tissue biopsy samples. Histopathology of cutaneous lesions (eg, keratinized foot pads) or frequently affected tissues (eg, lungs, bladder, and lymph tissue) can identify characteristic intracytoplasmic or intranuclear viral inclusions.^1^, ^2^ The turnaround time for these test results, when samples are collected antemortem, can delay appropriate treatment. Identifying additional diagnostic or clinical criteria that support a diagnosis of distemper could facilitate diagnosis and appropriate treatment earlier in the course of disease, especially in cases of young animals with suspicion of an infectious disease.

In a previous study,^3^ CDV antigen was demonstrated in long bone metaphyses (ie, humerus, radius, ulna, femur, tibia, and fibula) between 5 and 36 days after infection. Additionally, homogenous band‐like increased density of bone (sclerosis) was identified grossly just beneath the growth plate. Lesions in that study were histopathologically characterized by necrosis of osteoclasts, osteoblasts, and marrow cells as well as by persistence of primary spongiosa.^3^ These features are different from the previously described disease process of metaphyseal osteopathy, formerly referred to as hypertrophic osteodystrophy (HOD). Metaphyseal osteopathy histopathologically is characterized by a band of neutrophilic inflammation and necrosis within the primary spongiosa that can progress to lysis and infarction and, in chronic cases, periosteal and extraperiosteal woven bone proliferation at the metaphyses and physes.^4^, ^5^

To our knowledge, the identification of sclerosis secondary to CDV using radiography has not been described in the literature. Our objective was to describe the radiographic appearance of metaphyseal sclerosis secondary to canine distemper virus in 4 juvenile dogs and its distinction from metaphyseal osteopathy.

## 2 MATERIALS AND METHODS

Cases were selected based on a combination of available radiographic imaging that included a diagnosis of symmetric metaphyseal sclerosis of at least 1 long bone, a constellation of clinical signs (including neurologic signs if present) compatible with distemper, and diagnosis of CDV either by RT‐PCR or histopathologic identification of viral inclusions with or without immunohistochemistry. Cases 1 and 2 were presented to the University of Tennessee Small Animal Hospital through the emergency department. Cases 3 and 4 were presented to University of California, Davis Veterinary Medical Teaching Hospital emergency service. All radiographs were evaluated by board‐certified veterinary radiologists. Necropsies for dogs 1 and 2 were overseen and performed by board‐certified veterinary pathologists. Details of diagnostic test results and treatment were retrospectively acquired by review of medical records. Testing for CDV in all cases was performed at the respective institutions' in‐house diagnostic laboratories.

## 3 CLINICAL CASE REPORTS

### 3.1 Case 1

A 16‐week‐old female intact Giant Schnauzer was referred to the University of Tennessee Small Animal Hospital emergency service for further diagnostic testing and treatment after muscle tremors and 3 generalized tonic‐clonic seizures within 24 hours, managed by administration of diazepam before referral. The dog had been acquired from a California breeder 19 days before presentation and had reportedly been “partially vaccinated.” According to the owners, the dog had a cough since being acquired but was otherwise eating and drinking normally. Approximately 5 days before presentation to the emergency service, the dog had been brought to its primary veterinarian for lethargy and anorexia, and was subsequently diagnosed with aspiration pneumonia on thoracic radiographs, hospitalized overnight, and treated with, amoxicillin‐clavulanate potassium (Clavamox, Zoetis, Inc, Kalamazoo, Michigan).

At presentation, the dog was stuporous and physical examination abnormalities included a temperature of 102.1°F (reference interval, 100.6‐102.6°F) and increased respiratory rate of 68 breaths/min (reference interval, <40 breaths/min). Packed cell volume (PCV) was 35% (reference interval, 37%‐55%) and blood pressure was 70 mm Hg (noninvasive Doppler measurement; reference interval, 80‐150 mm Hg). Blood pressure was remeasured shortly after presentation and was normal at 110 mm Hg. A few hours after presentation, the dog had a generalized tonic‐clonic seizure which improved with treatment (levetiracetam, Hikma Pharaceuticals USA, Inc, Berkeley Height, New Jersey; midazolam, Alamaject, Inc, Morristown, New Jersey), but postictal signs and dull mentation persisted.

**FIGURE 1:**
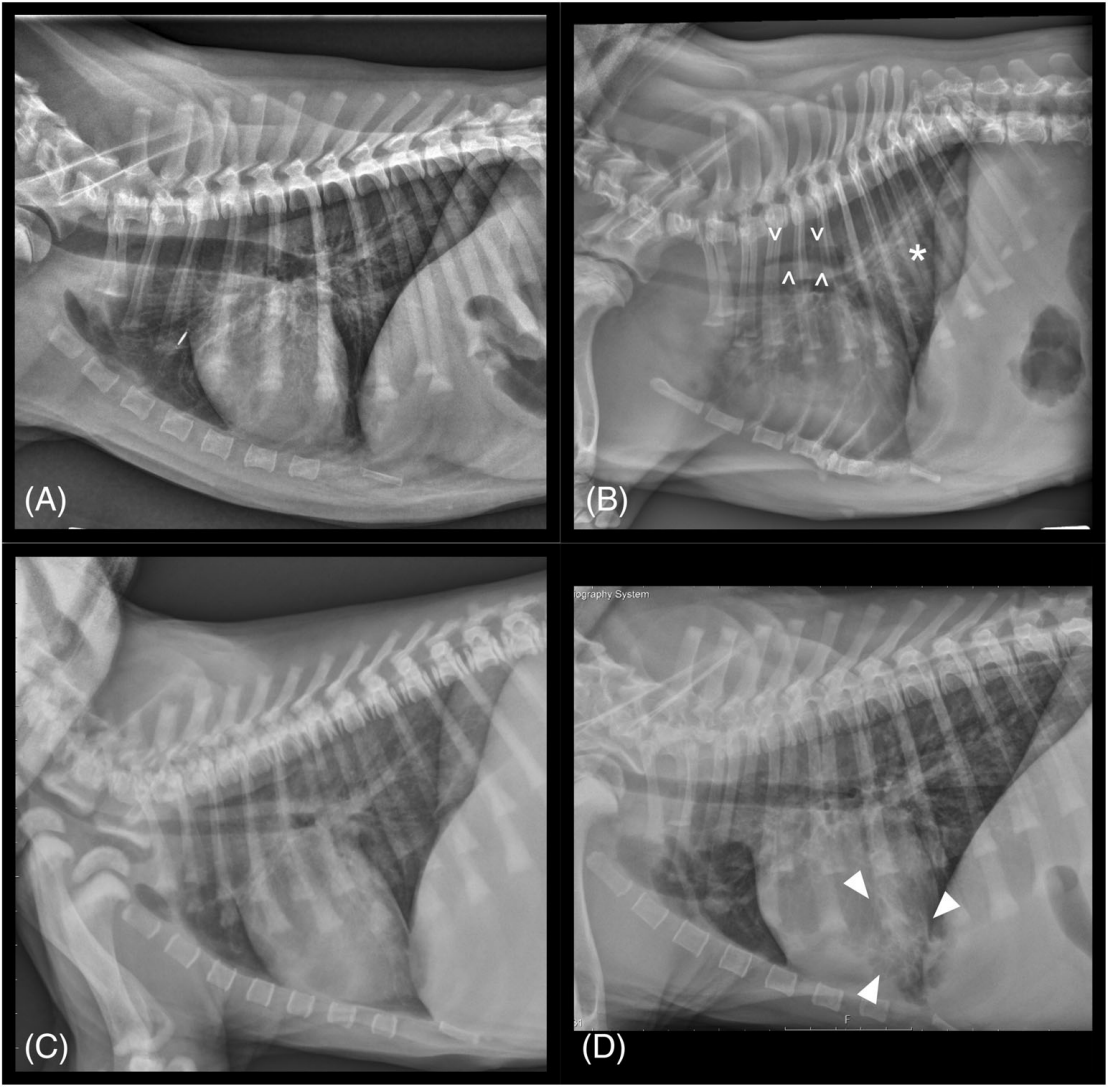
Lateral radiographs (all in left lateral recumbency) of Cases 1 through 4 showing diffuse mild unstructured interstitial pattern throughout the pulmonary parenchyma. (A) Case 1 has a generalized unstructured interstitial pattern throughout the lungs, more conspicuous in the caudodorsal lung fields and superimposed with the cardiac silhouette. There is a microchip within the lateral thoracic soft tissues, superimposed with the cranial thorax. (B) Case 2 generalized unstructured interstitial pattern in a brachycephalic patient. The patient's thoracic cavity is shortened and cardiac silhouette appears widened because of breed‐related conformation. There is evidence of a sliding hiatal hernia in caudodorsal thorax (asterisk), as well as mild gas dilation of the mid thoracic esophagus (carets). There are also caudal thoracic vertebral malformations, considered an incidental breed‐related finding. (C) Case 3 generalized unstructured interstitial pattern throughout the lungs, more conspicuous in the caudodorsal lung fields. (D) Case 4 diffuse mild bronchial and unstructured interstitial pulmonary pattern, and ventrally dependent, focal, marked unstructured interstitial pattern (arrowheads) superimposed with the heart apex

**FIGURE 2:**
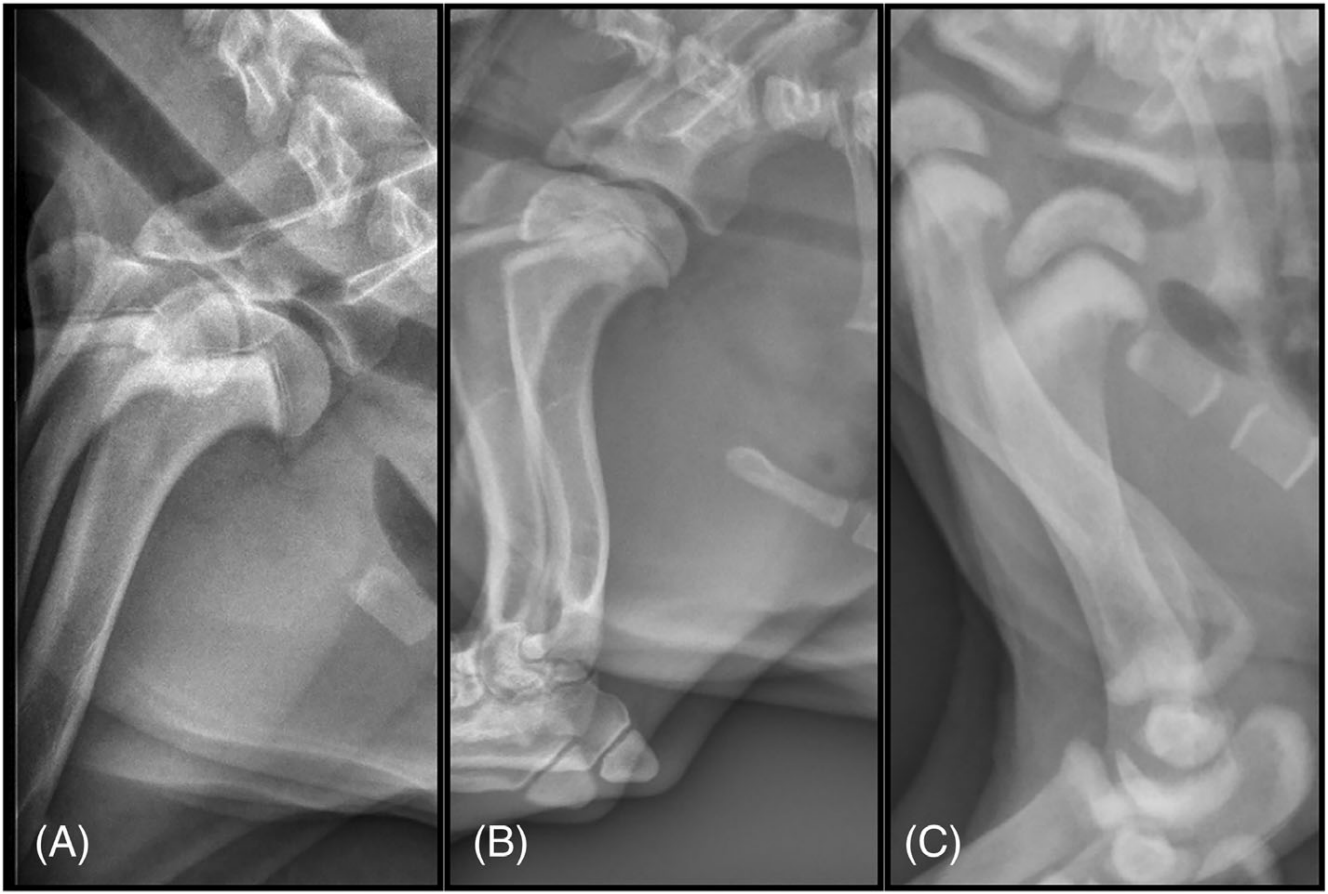
Lateral radiographs of Cases 1 through 3 showing the proximal humeral metaphyseal sclerosis on the edge of thoracic radiographs acquired of each patient. (A) Case 1, (B) Case 2, (C) Case 3. In (C), additional sites of sclerosis are identified on the edge of the thoracic radiograph; including the glenoid cavities, distal humeral metaphyses, proximal ulnae and proximal radial head

Thoracic radiographs acquired on presentation disclosed a diffuse mild unstructured interstitial pulmonary pattern, considered excessive for the patient's young age (Figure 1A). Additionally, symmetrical well‐defined sclerosis of the included proximal humeral metaphyses was noted. The proximal humeral physes themselves were considered normal with no evidence of lysis or periosteal proliferation (Figure 2A).

The dog was treated with crystalloid fluids, antibiotics, and antiseizure medications as deemed appropriate by the supervising clinician. Because of persistent muscle tremors, persistent dull mentation, recumbency, and lack of improvement the next day, the owners elected euthanasia. A distemper RT‐PCR assay on submitted urine was negative. A necropsy was performed.

**FIGURE 3:**
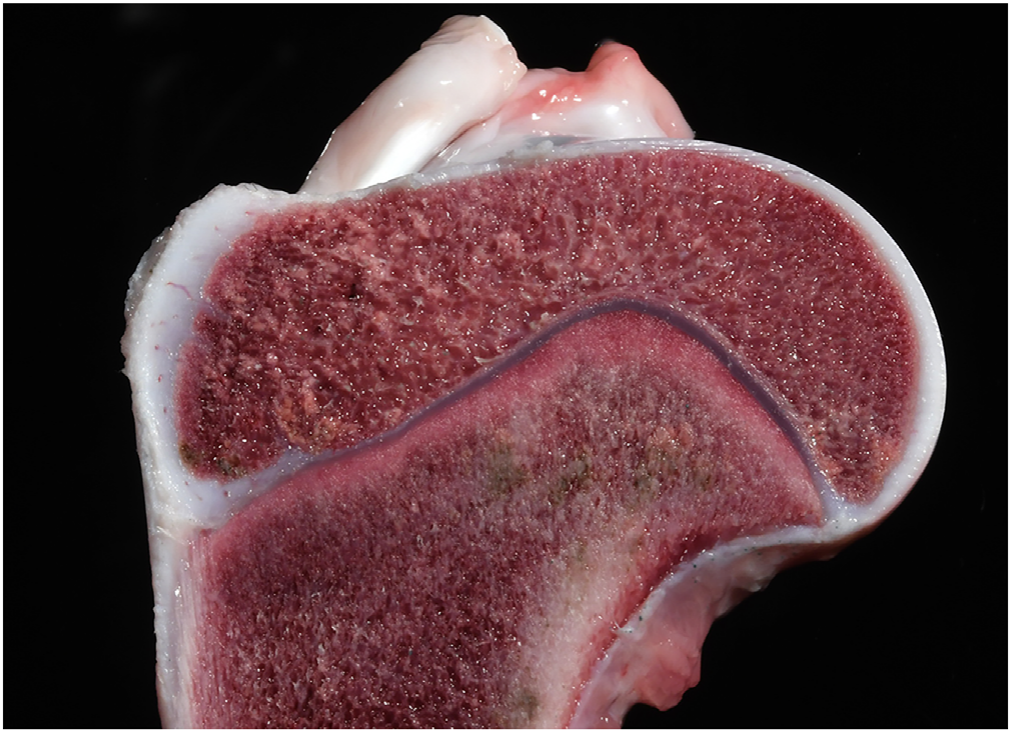
Gross photograph of cut section of proximal humerus showing a discrete band of pink‐white osteosclerosis of the metaphysis immediately adjacent to the physis

**FIGURE 4:**
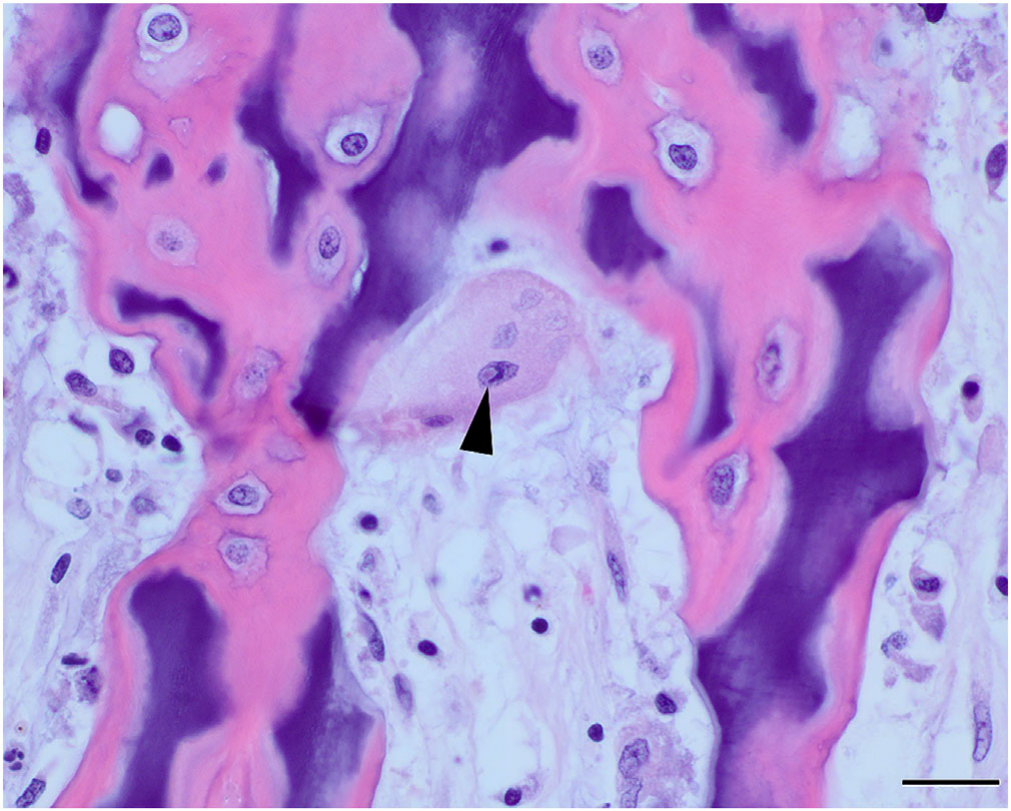
Photomicrograph of metaphysis of proximal humerus showing an osteoclast with faded (karyolytic) nuclei and an intranuclear inclusion (arrowhead). H&E stain; bar = 20 μm

**FIGURE 5:**
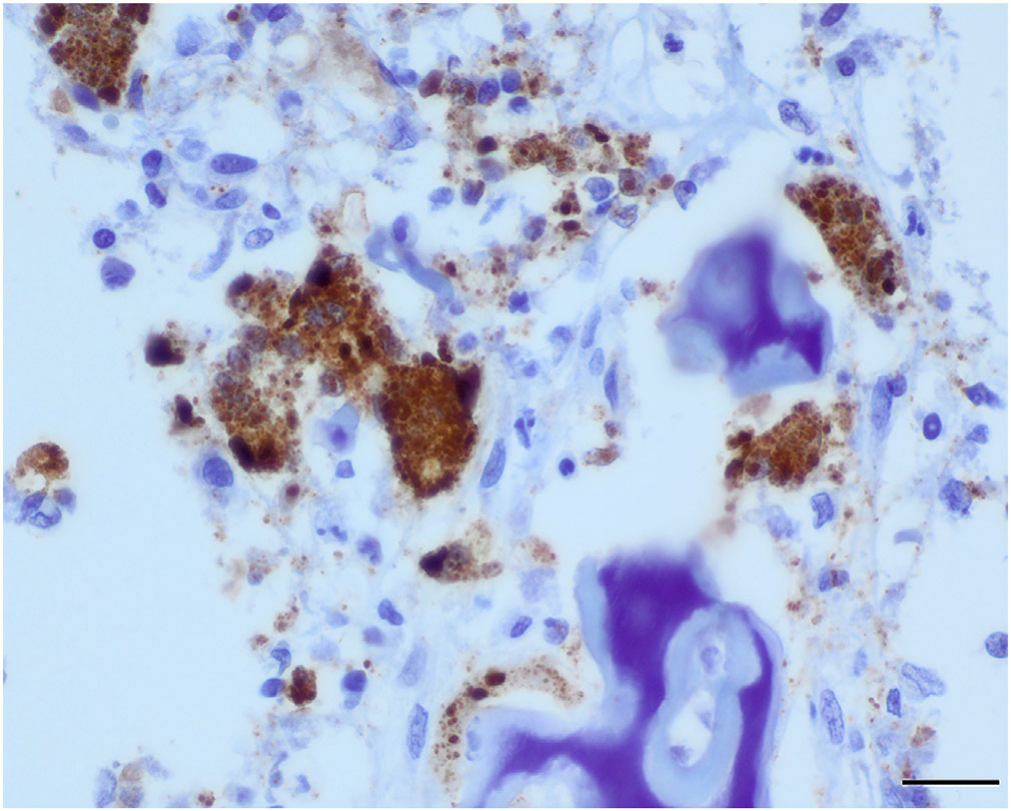
Photomicrograph of metaphysis of proximal humerus showing strong positive labeling (brown) of osteoclasts and osteoblasts for CDV antigen. CDV‐specific immunohistochemical reaction; bar = 20 μm

Necropsy identified opaque, pink to white, linear bands of hard, dense, sclerotic bone, 1 to 2 mm wide in the proximal and distal metaphyses of the long bones of both pelvic and thoracic limbs (Figure 3). The sclerotic lesions were parallel to the physes and spanned the full width of the affected bone. Histologically, throughout the long bone metaphyses, osteoblasts, osteoclasts and hematopoietic cells were hypereosinophilic with pyknotic, faded, or absent nuclei, consistent with necrosis. Some osteoclasts had small eosinophilic intranuclear or intracytoplasmic inclusions, or both (Figure 4). The primary spongiosa was retained, forming a thick band of metaphyseal sclerosis. Immunohistochemistry for CDV antigen was positive in osteoclasts and osteoblasts (Figure 5).

Grossly, the lungs were mottled dark red to purple. Eosinophilic intracytoplasmic and intranuclear inclusions were identified in bronchiolar epithelium, and immunohistochemistry for CDV antigen was positive throughout the lung, as well as in the urinary bladder epithelium. Reverse transcription‐PCR for CDV was negative on a sample of lung. Mild multifocal lymphoplasmacytic meningoencephalitis affected the mesencephalon and medulla oblongata. A section of pons and cerebellum was negative for rabies virus by direct fluorescent antibody test.

### 3.2 Case 2

Nine days after presentation of case 1, another puppy from the same household (a 4‐month‐old female intact French Bulldog) was presented to the University of Tennessee Small Animal Hospital for evaluation of progressive shaking and increased respiratory effort. This puppy had been acquired recently from a different breeder and also was reported to be “partially vaccinated.” The dog had been brought to the referring veterinarian a few days before for evaluation of lethargy. At that time, thoracic radiographs showed pulmonary infiltrates, and amoxicillin‐clavulanate potassium (Clavamox, Zoetis, Inc) was prescribed. The dog had initially improved on the antibiotics, but the owners noted progressive shaking and tremors over the 2 to 3 days before presentation.

At presentation, the dog was mildly dyspneic with increased respiratory rate and effort. Lung sounds were harsh bilaterally. On physical examination, the dog had mild muscle tremors but no other neurologic or muscular abnormalities. Other physical examination abnormalities included mildly increased heart rate of 126 beats/min (reference interval, 60‐120 beats/min) and increased respiratory rate of 84 breaths/min (reference interval, <40 breaths/min). The dog was admitted to the hospital and started on enrofloxacin injectable (Baytril, Elanco US, Inc, Greenfield, Indiana) and amoxicillin sodium sulbactam sodium injectable (Unasyn, New York, New York) and placed in oxygen at 40% FiO_2_ (fraction of inspired oxygen).

Thoracic radiographs the next day showed a generalized moderate unstructured interstitial pulmonary pattern (Figure 1B) and well‐defined metaphyseal sclerosis of the proximal humeral metaphyses with normal physes (Figure 2B). On CBC, leukopenia (WBC, 3.3 × 10^3^/μL; reference interval 4.7‐15.2 × 10^3^/μL) characterized by neutropenia was present. The dog was kept in oxygen and started empirically on IV antibiotics.

**FIGURE 6:**
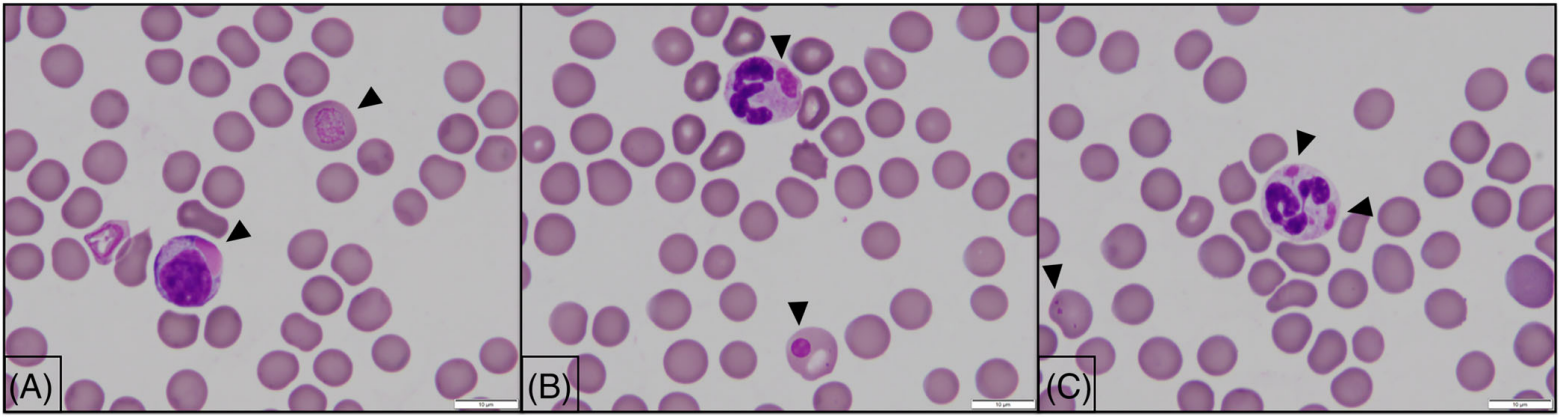
Microscopic images (100× objective) of peripheral blood smear from Case 2. (A) Image showing pink distemper inclusions in a lymphocyte and red blood cell (arrow heads). (B) Image showing distemper inclusions in a neutrophil and red blood cell. (C) Image showing distemper inclusions in a neutrophil and smaller distemper inclusions in a red blood cell

The dog's condition was relatively static over the first 24 to 36 hours of hospitalization and it was weaned off oxygen. During hospitalization, a urine CDV RT‐PCR test, blood lead concentration, and repeat CBC and serum biochemistry panel were performed. The repeat CBC showed pancytopenia (WBC, 3.3 × 10^3^/μL; reference interval 4.7‐15.2 × 10^3^/μL; platelets, 36 × 10^3^/μL; reference interval 147‐423 × 10^3^/μL; RBC, 3.95 × 10^3^/μL; reference interval 5.74‐8.64 × 10^3^/μL). Viral inclusions were identified on the blood smear in both red blood cells and neutrophils (Figure 6). Urine CDV RT‐PCR testing was negative and blood lead concentration was normal.

The dog's respiratory signs and laboratory abnormalities improved somewhat by the third day of hospitalization, and the muscle tremors were static. The owners elected to take the dog home on the fourth day of hospitalization. They returned the dog to the hospital the same evening because of deteriorating condition, particularly worsening respiratory distress. Repeat thoracic radiographs showed a progressively severe unstructured interstitial pattern diffusely affecting the lungs, with focal alveolar patterns in the cranioventral lung fields, raising concern for concurrent aspiration pneumonia. The owners elected euthanasia and necropsy.

At necropsy, the pleural cavity contained 15 mL of serosanguineous fluid, and the left and right cranial lung lobes were light red, firm to consolidated, and sank in 10% formalin. The remaining lung lobes were rubbery, failed to collapse, and also floated in 10% formalin. Hyperkeratosis of the paw pads was present. The thymus was approximately 50% smaller than expected. A diaphragmatic defect containing the cardia part of the stomach was present. On cut section, the humeral metaphysis had a band of opaque bone, grossly consistent with sclerosis.

Histologically, the lungs had diffuse lymphohistiocytic bronchointerstitial pneumonia and cranioventral aspiration pneumonia. Syncytial cells commonly contained round, eosinophilic, intranuclear, and intracytoplasmic inclusions. Throughout the brain, midbrain, and cerebellum, acute astrocytosis and gliosis were present with rare intranuclear and intracytoplasmic astrocytic inclusions. Moderate lymphoid necrosis was present in multiple organs. Epithelial cells in the peripheral cornea of the right eye contained viral inclusions and corneal edema was present. All pathologic findings were considered consistent with a final diagnosis of canine distemper.

### 3.3 Case 3

A 2½‐month‐old male intact Labrador mix was presented to the University of California, Davis Veterinary Medical Teaching Hospital for evaluation of seizures and upper respiratory clinical signs. The dog had been adopted 2 days before, had received its first vaccines, and since adoption the owners had noticed mucopurulent ocular discharge and serous nasal discharge. The day after adoption, the dog developed focal seizures characterized by left lip twitching and was brought to the referring veterinarian. Parvovirus fecal testing and fecal flotation were reportedly negative, and other laboratory testing reportedly was within normal limits. The puppy was treated with erythromycin 0.5% ophthalmic ointment (Bausch Health, Quebec, Canada) and amoxicillin‐clavulanate potassium (Clavamox, Zoetis, Inc) and referred for further evaluation.

The dog was presented the next day to the emergency service and the owners described an increased frequency of focal seizures and progressive lethargy. Physical examination abnormalities at presentation included bilateral mucopurulent ocular discharge, mild weight‐bearing lameness of the left thoracic limb, and multiple focal seizures during the examination (lasting approximately 15 seconds each). An RT‐PCR test for CDV of a conjunctival swab was positive, supporting a diagnosis of distemper. Plasma ammonia concentration was mildly increased at 86 μg/dL (reference interval, 0‐59 μg/dL) but was not considered indicative of a portosystemic shunt or hepatic dysfunction. The dog was started on phenobarbital (e5 Pharma LLC, Boca Raton, Florida) and doxycycline (liquid suspension, Chartwell Rx LLC, Mason, Ohio) PO; amoxicillin‐clavulanate potassium was discontinued.

The patient was discharged, but was re‐presented 1 week later for evaluation of progressive cough. The ocular discharge had improved somewhat, but the focal facial seizures persisted at a similar frequency. A CBC showed a macrocytic hypochromic regenerative anemia (HCT, 21%; reference interval, 40%‐55%) and leukopenia (3270/μL; reference interval, 6000‐13 000/μL), characterized by a lymphopenia (57/μL; reference interval, 1000‐4000/μL) and neutropenia (2161/μL; reference interval, 3000‐10 500/μL). Viral inclusions consistent with CDV were identified in erythrocytes and neutrophils on a blood smear. Thoracic radiographs showed a diffuse bronchial and unstructured interstitial pulmonary pattern (Figure 1C) as well as metaphyseal sclerosis of multiple bones, affecting the long bones most severely (Figure 2C). A Schirmer tear test showed inadequate tear production (6 mm/min, right eye; 11 mm/min, left eye; normal, >15 mm/min).

Amoxicillin‐clavulanate (Clavamox, Zoetis, Inc) PO was prescribed, and re‐evaluation in 1 week was recommended. The dog was returned to the emergency service 2 days later because of worsening respiratory distress and progressive neurologic signs. The owners elected euthanasia and but declined necropsy.

### 3.4 Case 4

An approximately 3‐month‐old male intact Belgian Malinois dog was presented to the University of California, Davis Veterinary Medical Teaching Hospital for vomiting and diarrhea. The puppy had been obtained from a breeder 3 days before presentation, had received 1 set of vaccines, and had been treated for gastrointestinal parasites. At presentation, the dog was tachycardic (220 beats/min; reference interval, 60‐120 beats/min) and dehydrated. A parvovirus test was negative. The owners declined hospitalization and elected outpatient treatment.

The puppy was re‐presented on emergency 2 weeks later for lethargy, coughing or retching, and left thoracic limb lameness. It had been taken to the referring veterinarian 3 days before and thoracic radiographs reportedly showed a diffuse bronchial pulmonary pattern and mild interstitial pulmonary pattern ventrally. Antibiotics were prescribed by the referring veterinarian and the dog reportedly temporarily improved before re‐presenting to UC Davis Emergency Service for worsening clinical signs, including left thoracic limb lameness. Upon presentation the second time, physical examination abnormalities included mild hyperthermia of 102.7°F (reference interval, 100.6‐102.6°F), bilateral ocular and nasal mucoid discharge, hyperkeratosis of the foot pads and nasal planum, harsh lung sounds with mildly increased respiratory effort, and lameness of thoracic left limb that could not be localized to a specific site. Head tremors and facial twitching were noted at initial presentation to the emergency service but not on follow‐up physical examinations after admission.

**FIGURE 7:**
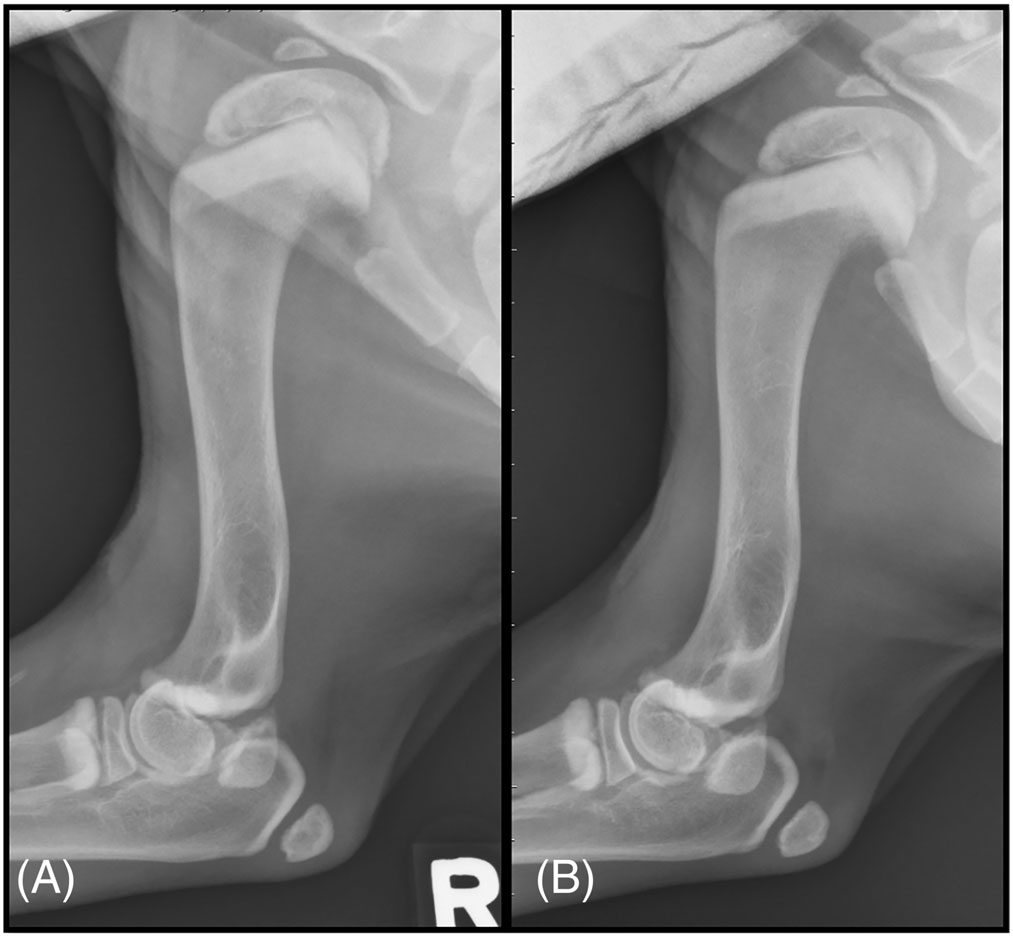
Lateral radiographs of the forelimbs of Case 4, (A) is a lateral radiograph of the right forelimb and (B) is a lateral radiograph of the left forelimb. Well‐defined metaphyseal sclerosis is noted most severely affecting the proximal humeral physes, but is also present at the distal humeral physes, proximal radial heads, and proximal ulnae

Abnormalities on CBC included severe lymphopenia (82/μL; reference interval, 1000‐4000/μL) and mild anemia (HCT, 28.6%; reference interval, 40%‐55%). Thoracic radiographs showed a diffuse mild bronchial and unstructured interstitial pulmonary pattern, and ventrally dependent, focal, marked unstructured interstitial pattern superimposed with the heart apex (Figure 1D). Lateral views of the proximal thoracic limbs showed multifocal, well‐defined, symmetrical sclerosis of the included metaphyses of the humeri, radii and ulnae bilaterally (Figure 7). Irregular margination was noted of the humeral heads, as well as on the scapular and ulnar apophyses. An RT‐PCR test for CDV of a conjunctival swab was positive, supporting a diagnosis of distemper. Schirmer's tear test measured 9 mm/min in both eyes (reference interval, >15 mm/min), supportive of keratoconjunctivitis sicca likely secondary to CDV. The dog was discharged on doxycycline tablets 50 mg PO BID (Heritage Pharmaceuticals, Edison, New Jersey) and eye lubricant (Optixcare, Aventix, Burlington, Ontario). The owners were advised to have the dog reevaluated by their primary veterinarian, but the dog was lost to follow‐up.

## 4 DISCUSSION

In this report, all 4 patients had metaphyseal sclerosis of long bones identified on radiographs and diagnosis of canine distemper. The presence of CDV antigen on immunohistochemistry of the metaphyseal sclerosis lesions in case 1 suggests the virus may have caused the lesions. Sclerosis associated with CDV is distinct from the well‐described, usually self‐limiting, metaphyseal osteopathy. Metaphyseal osteopathy is a disease of young growing dogs, mainly those of large and giant breeds, usually 3 to 4 months of age, and most often affects the distal radius and ulna.^6^, ^7^ It has been anecdotally reported to be associated with CDV vaccines.^4^, ^8^ The cases in our report do not display the radiographically aggressive changes described in metaphyseal osteopathy, characterized by bands of lysis affecting the metaphyses and parallel to the physes (frequently referred to as the “double physis sign”).^9^, ^10^ Additionally, there was no osseous proliferation extending around the metaphysis or physis, which also has been described in metaphyseal osteopathy, usually when its more chronic.^9^

Two prior studies have described metaphyseal lesions histologically in dogs with CDV infection. In 1 study,^3^ lesions were characterized histopathologically by necrosis of osteoclasts, osteoblasts and marrow cells as well as by persistence of primary spongiosa, as seen in case 1 of this report. This finding is distinct from metaphyseal osteopathy, which is characterized by persistence of the mineralized cartilage lattice of the primary spongiosa and intense intertrabecular suppurative inflammation with necrosis and disappearance of osteoblasts.^4^ An earlier study^11^ found evidence of prior CDV infection in metaphyseal marrow cells, osteoblasts, osteocytes and particularly osteoclasts of 2/4 dogs that had recovered from CDV infection.

The long bone metaphyseal changes described in our report have a similar appearance to the previously described metaphyseal lesions formed secondary to lead toxicity (seen in both humans and animals), but blood lead concentration was measured in 1 of the dogs and was normal. The radiographic osseous lesions secondary to lead poisoning are described as radiopaque lines or bands within the metaphyses, adjacent to the growth plates.^12^, ^13^, ^14^ Lead precipitates as insoluble salts in bone and represents a type of long‐term storage in the bone.^12^ The clinical signs of lead poisoning include neurologic and gastrointestinal signs, which overlap with clinical signs of CDV, and therefore a thorough history and physical examination should be performed to evaluate the possibility of lead poisoning when metaphyseal sclerosis is identified radiographically.

Canine distemper is an important differential diagnosis for nonvaccinated or incompletely vaccinated dogs that display clinical signs of infectious disease. Patients with suspected canine infectious respiratory disease complex or parvovirus infection should have distemper as a differential diagnosis because clinical signs of CDV can sometimes mimic these other infectious diseases of young animals. Canine distemper virus has a very broad geographic distribution and, although it most commonly affects dogs and other canids (such as coyotes, foxes, and wolves), other species have been affected, including mustelids (such as ferrets, skunks, and otters), and large wild feline species.^1^, ^2^

Two of the patients in our study were reported to have unilateral lameness, which is in contrast to the previous case series of 42 dogs in which no dogs were reported to have clinical signs related to the osseous lesions.^3^ Two of the dogs in our study had a toe‐touching (case 4) or weight‐bearing (case 3) lameness, both of the left thoracic limb. Radiographically, the osseous abnormalities symmetrically affected the thoracic limbs and a cause for the localized lameness was not identified. It is possible that skeletal pain was associated with the changes, but it is also possible the lameness was weight‐shifting, associated with soft tissue abnormalities, or involved a portion of the left front limbs that was not imaged.

Clinically, the most commonly utilized tests for diagnosing CDV include RT‐PCR, which can be performed on various body fluids, or histopathology using necropsy specimens or skin biopsy samples (such as from the footpad or nasal planum).^1^ Immunofluorescence of tissue or fluid specimens has the limitation of only being detectable within 3 weeks after infection while the virus is still in epithelial cells.^2^, ^15^ Immunohistochemistry confirmed the diagnosis of CDV in case 1, and necropsy with histopathology was helpful in confirming the diagnosis of CDV for both case 1 and its housemate. Polymerase chain reaction is considered 1 of the most efficient tests compared to others, and is advantageous because of its high sensitivity and the different types of samples it can be performed on, including urine.^15^ Cases 1 and 2 were confirmed as having CDV based on gross and microscopic necropsy findings as well as viral inclusions seen antemortem on a blood smear (case 2). However, both dogs had a negative antemortem urine RT‐PCR test results. Although the sensitivity of RT‐PCR for CDV is high, it is possible that viral nucleic acid was no longer present in the urine submitted, thus creating a negative result. The lung sample taken at necropsy of dog 1 (which had viral inclusions microscopically and viral antigen by immunohistochemistry) also had negative RT‐PCR for CDV. This result may have been a false negative. The laboratory was quality certified through the Veterinary Laboratory Association and reportedly utilized positive and negative controls. It is possible that a false negative could have resulted from presence of PCR inhibitors, a virus containing mutations at the sites targeted by the primers, or improper storage of the sample.

False positive RT‐PCR results sometimes may occur in patients that have been vaccinated recently using modified live virus (MLV) vaccines. The MLV vaccine can be detected in blood, urine, and swabs of body fluids because RT‐PCR test is highly sensitive. Quantitative RT‐PCR can potentially better distinguish vaccine interference from infection by detecting the much higher amount of virus shedding seen in infection. However, in acute infection, shedding may be low and thus mimic the viral load seen after recent vaccination. A real‐time RT‐PCR test that distinguishes wild‐type CDV strains from vaccine strains is an option when trying to differentiate the 2.^16^ In our case series, the patients had a mixture of incomplete and questionable vaccination, and thus any relationship between their clinical and laboratory findings with their vaccine status could not be explored. A false positive is considered unlikely in case 3 because of identification of viral inclusions on a blood smear. In cases 3 and 4, RT‐PCR tests were performed at the laboratory of their institution, where appropriate, established, quality control measures were utilized and therefore contamination or other handling errors that could cause false positives are considered unlikely. Vaccination as a cause for a false positive result in case 4 cannot be ruled out because the patient reportedly received 1 set of vaccines but details were not provided. False positive results for both cases 3 and 4 are considered unlikely because the clinical findings were supportive of CDV.

Cases 2 and 3 had viral inclusions identified on blood smears, strongly supporting a diagnosis of CDV. Intracytoplasmic distemper viral inclusions on blood smear are specific for the disease, but are only detected in the early phase of the disease and may be in low numbers. Therefore, their absence on a blood smear does not rule out CDV infection.^17^

In our study, all patients had clinical signs of respiratory disease, as well as an abnormal diffuse bronchial and interstitial pulmonary pattern on radiographs. Dog 4 had a more focal marked unstructured interstitial pattern ventrally, which may have represented bronchopneumonia or aspiration pneumonia. Viral pneumonia caused by CDV and potential secondary bronchopneumonia caused by bacterial infection are well‐known and documented, explaining these patients' clinical signs. Dog 2, a French bulldog, was confirmed at necropsy to have an additional complication of aspiration pneumonia, which was suspected clinically because of a ventrally distributed alveolar pattern seen on follow‐up radiographs in addition to the diffuse interstitial pulmonary pattern. Hiatal hernia, diagnosed at necropsy, may have caused regurgitation, predisposing dog 2 to aspiration pneumonia.

Necropsy of dog 2 identified a small thymus and multifocal lymphoid depletion microscopically throughout organs. This finding is consistent with lymphocytic viral‐induced apoptosis, as previously reported in lymphocytes within blood, bone marrow, thymus, spleen, and other organs.^1^, ^17^, ^18^ This mechanism also explains the leukopenia noted in cases 2, 3, and 4.

Treatment for CDV is typically supportive and directed at managing clinical signs. In our study, 3 of the 4 dogs were euthanized because of the severity of clinical signs, and the 4th dog was lost to follow‐up. It is likely that mildly symptomatic dogs with distemper may go undiagnosed if veterinary care is not sought or the response to initial supportive treatment is sufficient.

Neurologic abnormalities may occur in up to 30% of infected dogs, and can occur weeks after the onset of clinical signs or recovery from systemic illness.^1^, ^17^ Neurologic signs also can develop later in life.^1^, ^17^ All dogs in our report had neurologic signs, ranging from mild muscle tremors to debilitating tonic‐clonic seizures. Dogs 3 and 4 had decreased tear production, consistent with keratoconjunctivitis sicca, previously described as a possible sequela to distemper and hypothesized to be caused by damage to the lacrimal glands.^1^

We describe a previously unpublished radiographic finding of metaphyseal sclerosis associated with CDV infection. Our report indicates that metaphyseal sclerosis may support the diagnosis of CDV infection in young dogs suspected of infectious disease. Furthermore, the sclerosis identified secondary to CDV is distinct from the necrosis and lysis of metaphyseal osteopathy.

## CONFLICT OF INTEREST DECLARATION

Authors declare no conflict of interest.

## OFF‐LABEL ANTIMICROBIAL DECLARATION

Authors declare no off‐label use of antimicrobials.

## INSTITUTIONAL ANIMAL CARE AND USE COMMITTEE (IACUC) OR OTHER APPROVAL DECLARATION

Authors declare no IACUC or other approval was needed.

## HUMAN ETHICS APPROVAL DECLARATION

Authors declare human ethics approval was not needed for this study.
